# Syndromic Surveillance of Communicable Diseases in Mobile Clinics During the Arbaeenia Mass Gathering in Wassit Governorate, Iraq, in 2014: Cross-Sectional Study

**DOI:** 10.2196/10920

**Published:** 2019-10-07

**Authors:** Faris Lami, Wejdan Asi, Adnan Khistawi, Iman Jawad

**Affiliations:** 1 Department of Community and Family Medicine College of Medicine University of Baghdad Baghdad Iraq; 2 Wasit Directorate of Health Iraq Ministry of Health Wasit Iraq; 3 Directorate of Public Health Iraq Ministry of Health Baghdad Iraq

**Keywords:** Arbaeenia, mass gathering, syndromic surveillance, communicable diseases, Iraq

## Abstract

**Background:**

Arbaeenia is the largest religious mass gathering organized annually in Karbala city, Iraq, and is attended by 8-14 million people. Outbreaks of communicable diseases are a significant risk due to overcrowding and potential food and water contamination. Syndromic surveillance is often used for rapid detection and response to disease outbreaks.

**Objective:**

This study was conducted to identify the main communicable diseases syndromes among pilgrims during the Arbaeenia mass gathering in Wassit governorate, Iraq, in 2014.

**Methods:**

This cross-sectional study was conducted in the 40 mobile clinics established within Wassit governorates along the road to Karbala during the Arbaeenia mass gathering. Six communicable disease syndromes were selected: acute watery diarrhea, bloody diarrhea, fever and cough, vomiting with or without diarrhea, fever and bleeding tendency, and fever and rash. A simple questionnaire was used to directly gather basic demographics and the syndromic diagnosis from the attendees.

**Results:**

A total of 87,865 patients attended the clinics during the 10-day period, with an average of 219 patients/clinic/day. Approximately 5% (3999) of the attendees had communicable diseases syndromes: of these, 1693 (42%) had fever and cough, 1144 (29%) had acute diarrhea, 1062 (27%) presented with vomiting with/without diarrhea, and 100 (2%) had bloody diarrhea. The distribution of the syndromes did not vary by age or gender. Stool specimen cultures for *Vibrio cholerae* performed for 120 patients with acute diarrhea were all negative.

**Conclusions:**

Syndromic surveillance was useful in determining the main communicable diseases encountered during the mass gathering. Expansion of this surveillance to other governorates and the use of mobile technology can help in timely detection and response to communicable disease outbreaks.

## Introduction

Mass gatherings are defined as preplanned public events that are held for a limited time period and attended by more than 25,000 people. Mass gatherings can be festivals, religious activities, sporting events, concerts, political rallies, or other events. The duration of the event could range from several hours to several days [1].

Mass gatherings create favorable conditions for infectious disease transmission [2]. Participants in mass gatherings are at risk of infection, with the potential to transmit diseases to their local communities after returning from the event. Mass gatherings may also exacerbate noncommunicable disease conditions, which may lead to emergencies and hospital admissions [3]. Infectious disease outbreaks are common during mass gatherings [4-6]. Severe respiratory infections are major causes of hospitalizations during mass gatherings such as Hajj [7-10]. National surveillance systems are often incapable of monitoring the public health threats from mass gatherings [11]. Although mass gatherings pose challenges to health care systems worldwide [12], they provide an opportunity to strengthen both emergency and routine surveillance and response systems, which can benefit countries long after the event [13].

Several religious mass gatherings occur on an annual basis in Iraq, drawing up to 10 million pilgrims at a time. The mass gathering known as “Arbaeenia” is one of the largest annual mass gatherings in the world, during which individuals may walk up to 600 km through Iraqi governorates to visit the Imam Hussein’s shrine in Karbala to commemorate the 40th day after his martyrdom [14].

The use of appropriate surveillance systems ensures timely information management for effective planning and response to infectious diseases threats during the pilgrimage [15]. The influx of people places a strain on existing surveillance and response systems, and due to the media and political attention generated by mass gatherings, the adverse consequences of any negative health event may be greatly magnified [13].

As part of the health services delivered to pilgrims, Iraq’s Ministry of Health set up mobile clinics along the main roads leading to Karbala to provide ambulatory health services to the pilgrims. Public health surveillance during mass gatherings is essential to detect and respond to disease epidemics that could have pandemic potential, in a timely manner. Syndromic surveillance makes preliminary clinical information available in a much more timely manner and avoids a potentially lengthy delay required for definitive, laboratory-confirmed diagnoses.

Currently, the routine national surveillance system for the main communicable diseases in Iraq is not implemented in the temporary mobile clinics during mass gatherings events, as these clinics are more concerned with provision of basic curative services. To fill the gap in disease surveillance in these clinics, we introduced the syndromic surveillance that covers a spectrum of communicable diseases of concern to the national public health system during mass gatherings.

The objective of this study was to identify the main communicable disease syndromes reported among mass gathering attendees who seek care from the mobile clinics in Wassit governorate during the Arbaeenia mass gathering event. Information obtained from this study is expected to enhance future planning of mass gathering events and develop an effective surveillance system for the mass gatherings.

## Methods

We conducted this cross-sectional study during the Arabeenia mass gathering that occurred on December 1-10, 2014, in Wassit governorate, Iraq. Wassit governorate is located in the middle of Iraq. Kut city is the center of the governorate, 180 km south of the capital Baghdad. The area of Wassit governorate is 17,012 km^2^, and the population is 1,240,930 people.

The study was conducted in the 40 mobile clinics that were set up in Wassit governorate along the 140-km road from the southern borders of Wassit, with Misan governorate to the northern borders along Babylon governorate. Throughout the country, these mobile clinics are established during the mass gathering event and provided only ambulatory curative services to the pilgrims. Only a small proportion of these clinics are run by medical doctors.

In this study, a basic communicable disease surveillance system was started in these 40 clinics after obtaining official approval from Iraq’s Ministry of Health. Six communicable disease syndromes were selected: acute watery diarrhea, bloody diarrhea, fever and cough, vomiting with or without diarrhea, fever and bleeding tendency, and fever and rash.

A short form was developed to gather data from all patients who attended these mobile clinics and presented with any of the six syndromes. The form included the age (classified only in two categories: <5 years and >5 years), sex, the total number of patients attending the mobile clinic, and the diagnosis of communicable diseases classified into the six syndromes listed above. The information in the form was recorded in the mobile clinic’s registry book. All the health workers working in these clinics received a short training by the communicable disease surveillance officers in Wassit governorate on how to fill the form and call the surveillance officers when needed, based on the guidelines of the Iraq Communicable Diseases Control Centre.

Data entry and analysis were performed using Epi Info 7 software (Centers for Disease Control and Prevention, Atlanta, Georgia). Frequency distribution of different communicable disease syndromes and their distribution by age, sex, and days of data collection was performed.

## Results

A total of 87,865 patients attended the mobile clinics in the 10-day period when the pilgrims walked through Wassit governorate on their way to Karbala city. The number of average daily attendees was 8786 patients, with 219 patients/clinic/day. There were 3999 patients with different communicable disease syndromes, constituting 4.6% of the total patients who attended the mobile clinics. Table 1 shows the number and proportion of patients who attended the clinic for the selected communicable disease syndromes.

Approximately 2% (1693/87,865) of the pilgrims attended the clinics for fever and cough, 1.3% (n=1144) attended for acute diarrhea, and 0.11% (100/87,865) attended for bloody diarrhea. During the 10 days of observation, none of the attendees to the mobile clinics presented with fever and rash or fever and bleeding syndromes. Moreover, 120 (10.5%) stool samples were randomly collected from patients with acute diarrhea for culture of *Vibrio cholerae*, all of which tested negative.

Fever and cough constituted the major proportion of communicable diseases presented at the mobile clinics (1693/3999, 42.3%), followed by acute diarrhea (1144/3999, 28.6%), vomiting with or without diarrhea (1062/3999, 26.6%), and bloody diarrhea (100/3999, 2.5%).

The reported syndromes were almost similarly distributed between the two age groups and both sexes, except acute diarrhea, where 61% (699/1144) of the cases were reported among attendees aged>5 years (Table 2).

**Figure 1:**
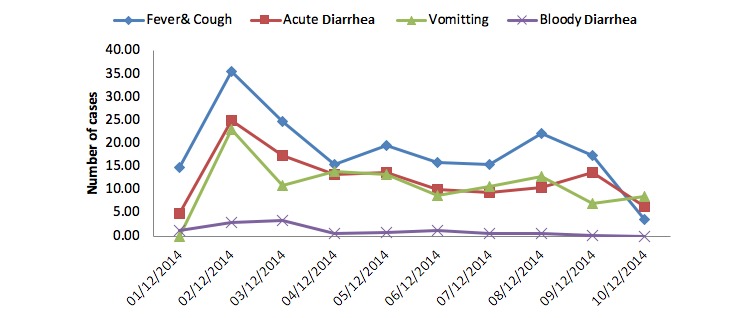
Distribution of the patients with reported communicable diseases syndromes over the 10-day study period in the Arbaeenia mass gathering in Wasit, Iraq, in 2014.

The time distribution of the four syndromes is displayed in Figure 1. The highest proportion of the four syndromes was reported on the second day. Apart from the small increase in the proportion of fever and cough and vomiting on the eighth day and acute diarrhea on the ninth day, there was a gradual decrease over the study days.

**Table 1:** Distribution of communicable disease syndromes among mobile clinics attendees during the Arbaeenia mass gathering in Wassit, Iraq, in 2014 (N=87,865).

Communicable disease syndrome	Total mobile clinic attendees, n (%)
Fever and cough	1693 (1.93)
Acute diarrhea	1144 (1.30)
Vomiting with or without diarrhea	1062 (1.21)
Bloody diarrhea	100 (0.11)
Fever and rash	0 (0.0)
Fever and bleeding	0 (0.0)

**Table 2:** Distribution of syndromes by sex and age groups at the Arbaeenia mass gathering in Wassit, Iraq, in 2014.

Syndrome	Gender		Age group		Total, n
	Male, n (%)	Female, n (%)	>5 years, n (%)	<5 years, n (%)	
Fever and cough	804 (47.5)	889 (52.5)	802 (47.4)	891 (52.6)	1693
Acute diarrhea	550 (48.1)	594 (51.9)	699 (61.1)	445 (38.9)	1144
Vomiting with or without diarrhea	502 (47.3)	560 (52.7)	502 (47.3)	560 (52.7)	1062
Bloody diarrhea	55 (55.0)	45 (45.0)	52 (52.0)	48 (48.0)	100
Total	1911 (47.8)	2088 (52.2)	2055 (51.4)	1944 (48.6)	3999

## Discussion

Despite the increasing number of people attending the Arbaeenia mass gathering, the preventive health services, particularly surveillance, of important diseases that harbor the risk of mortality and potential spread is far beyond that expected for this important mass gathering. This is the first study that highlighted an attempt to initiate communicable disease surveillance during this mass gathering using the syndromic approach. Syndromic surveillance was used in mass gatherings besides its use to detect outbreaks such as the H1N1 influenza pandemic [16,17].

The study focused on six important communicable disease syndromes, considering the country’s common disease context. The findings of the study highlighted the importance of syndromes of respiratory and gastrointestinal infections. Although these infections were expected, considering the exposure of the mass gathering attendees to the crowded and unhygienic environments, which create favorable conditions for transmission of respiratory and food borne diseases, the proportion of the attendees with these syndrome was less than that expected.

Apart from the Early Warning, Alert and Response Network surveillance system that uses the syndromic approach and is implemented in a number of camps for the internally displaced people in northern Iraq, the national surveillance system for communicable diseases in Iraq reported diseases rather than syndromes. This reduced the value of comparing our findings to the national figures of communicable disease surveillance.

Fever and cough syndrome is caused by many pathogens that are transmitted mainly from person to person through the respiratory tract, and it was the leading reported syndrome in this study. The Arbaeenia mass gathering in the year of the study took place during the wintertime, which could explain the high proportion of respiratory syndrome in the study.

Many pathogens including bacterial, parasitic, or viral pathogens can lead to acute diarrhea; the most dangerous pathogen of interest during mass gatherings is *V. cholerae*. Fortunately, all collected stool samples tested negative for *V. cholerae*. In a previous study conducted in the country, *Escherichia coli* and *Salmonella* species were the predominant causes of acute diarrhea [18]. Acute diarrhea and vomiting in the mass gathering could be related to an unhygienic environment of serving foods and drinks. All foods and drinks served to the pilgrims are freely provided by the local people, but they are all unlicensed by the health authorities, with minimal monitoring and auditing. In general, acute diarrhea diseases affected young children more than older adults; we found that the number of affected individuals aged>5 years was higher than that of children aged<5 years. Besides the narrow grouping of ages into only <5 years and >5 years, we did not have a denominator, and the mass gathering clinics only reported incident cases of these syndromes.

Fever and rash syndrome, which might indicate measles or rubella, were not reported in this study. This finding coincided with the fact that southern Iraq was free of measles and rubella in 2014. There were also no cases of fever and bleeding syndrome. In Iraq, the most common cause of hemorrhagic fever is the tick-borne Crimean-Congo hemorrhagic fever, a few cases of which are reported annually. The winter season does not support transmission of this disease.

The observed peak of the four syndromes on the second day of surveillance that immediately followed a very low proportion on the first day is more likely due to overattendance after launching the clinics rather than a coincident outbreak of the four syndromes together. The investigation performed later by the surveillance officers did not reveal any clustering of cases in particular mobile clinics.

We can conclude that the syndromic surveillance of communicable diseases, installed during mass gatherings can provide timely data on important communicable disease of concern to public health officials, which, in turn, can help in timely response. It is also useful in determining the main communicable disease syndromes and their profile. This can help in developing preparedness plans for the coming mass gatherings. Iraq’s Ministry of Health should to expand this surveillance to other governorates involved in the mass gathering event and upgrade it by using mobile technology that can help in having detailed data on different diseases and injuries and ensure a timely response.

The study has some limitations. The population at risk for communicable disease syndromes was not known; thus, the rates of syndromes could not be estimated. The age group>5 years clubs together people of a wide age range with varied risk, which limits the comparison of syndromes by age. Since this study was limited to Wassit governorate and 40 mobile clinics, it is difficult to generalize the results to all mobile clinics in the country that provided basic medical services to the Arbaeenia mass gathering attendees.
